# The ethnoecology of *Caiçara *metapopulations (Atlantic Forest, Brazil): ecological concepts and questions

**DOI:** 10.1186/1746-4269-2-40

**Published:** 2006-09-29

**Authors:** Alpina Begossi

**Affiliations:** 1 Fisheries and Food Institute, Rua Coronel Quirino 1636 apt.1, Campinas, 13025-002, S.P., Brazil & Museu de História Natural IB UNICAMP, Brazil

## Abstract

The Atlantic Forest is represented on the coast of Brazil by approximately 7,5% of remnants, much of these concentrated on the country's SE coast. Within these southeastern remnants, we still find the coastal Caiçaras who descend from Native Indians and Portuguese Colonizers. The maintenance of such populations, and their existence in spite of the deforestation that occurred on the Atlantic Forest coast, deserves especial attention and analysis. In this study, I address, in particular, the Caiçaras who live on the coast of São Paulo and Rio de Janeiro States, illustrating with examples of coastal inhabitants from other areas, such as Bahia State (NE coast) and of other forested areas (riverine caboclos of the Amazon). The major focus of this study, based on previous research, performed since 1986 in several populations or villages of the Atlantic Forest coast, is to understand the resilience of the Caiçaras, which is analyzed using ecological concepts, such as metapopulation, resilience and adaptive cycles. The Caiçara populations are located on islands (Búzios, Comprida, Grande, Ilhabela, Jaguanum, Gipóia) and on the coast (Bertioga, Puruba, Picinguaba, among others). Information gathered about the Caiçaras regarding the economic cycles of the local regions, along with ecological, historical and economic data available, are used to understand such resilience, and are complemented with comparative examples from the Brazilian Amazon and with variables such as the local restrictions imposed by environmental governmental agencies.

## Background

Concepts in ecology have been, for a long time, useful in order to understand processes within and between human populations, and their environment. Examples of the uses of these concepts are found in disciplines such as Ethnobiology or Human Ecology [1,2]. This study, based on previous fieldwork carried out along 20 years in the Atlantic Forest coast, actually represents a mental exercise with the aim of helping understanding the connection between Caiçara populations and their historical coevolution within their surroundings. For that enterprise, ecological concepts such as of metapopulation and resilience sounded suitable, bringing insights and still more questions about the future scenarios for the Atlantic Forest Coast and their native inhabitants, the Caiçaras.

## The Atlantic Forest

Originally, Brazilian Atlantic Forests covered 1.1 million km^2^, or 12% of the land surface of the country [3]. The Atlantic Forest remnants, currently about 7.5%, are an important area of endemism in Brazil [4], being composed of two major vegetation types: the Atlantic Rain Forest, located at low to medium elevations along the coast, and the semi-deciduous forest, located at elevations > 600 m in the interior [5]. The highest mean rainfall in the Atlantic Forest is found at the seaward side of the Serra do Mar, up to 3,600 mm [3].

In five centuries of occupation, the exploitation of the Atlantic Forest included pau brazil (*Ceasalpina echinata*), heart of palm (*Euterpe edulis*), xaxim (*Cyathea *sp.), and cultivation of sugar cane, coffee and cocoa (this last in Bahia State)[6]. The Atlantic Forest was cleared especially for timber, firewood, charcoal, agriculture, cattle ranching, and the construction of cities [5].

## The inhabitants of the Brazilian Atlantic Forest coast

The rural native inhabitants of the SE Atlantic Forest coast, descendants of Native Indians and Portuguese, are named 'C*aiçaras*'. Caiçaras descend from Tupinambá Indians, the first inhabitants of the Brazilian coast. Since the fifties, anthropologists and geographers have studied the life of the Caiçaras, such as their history and the economy in the region of São Sebastião Island [7] and Búzios Island [8], among other areas. Other authors have studied Caiçara communities in a variety of other aspects, such as their ethnology, economy, demography, technology, folk music and dance [9-11].

The ecology of the Caiçaras, such as their diet, food taboos, fishing, ethnoichthyology, and ethnobotany, as well as sea territoriality, has been published elsewhere [2,12-24]. The Caiçara diet is based on fish, rice and beans, manioc flour, and whenever possible, spaghetti. Just as for other Brazilians, rice and beans are an everyday food. Marine resources account for 40 to 70% of the animal items found in Caiçara meals.

Fishing, especially on the coastal sea, is usually performed in paddled or motorized canoes, and the gear used varies per locality. For example, at Búzios Island and at the Grande bay area, hook and line (including ripper jig) and set gillnets predominate; at Sepetiba bay, encircling nets for shrimp and fish are gear used. Fish traps are also employed, such as the two different cercos (fish traps) used on the southern Brazilian coast. The first, brought by the Japanese migrants in the thirties (Japanese kaku-ami), is made of chambers of nets; the second is a local trap made with bamboo, used especially for mullets and snooks [25]. Information on these fisheries has been already published [12-16,27-31].

Since the fifties, a general trend among the Caiçaras has occurred. They have shifted their source of cash from agricultural products (especially manioc flour, *Manihot esculenta*) to fishing, probably due to a general price decrease for agricultural products [29]. In addition, the processing of manioc, producing manioc flour, is labor-intensive, yielding lower returns compared to fishing. Its production involves peeling, grinding, pressing (to remove the cyanidric acid) and toasting [17]. Currently, there are especially two cash related economic activities in the communities studied: fish commercialization and tourist-related activities. The latter activity include housekeeping, house renting to tourists, sight-seeing trips, fishing conducted by local fishers, and handicraft that is locally sold. Fish, mostly landed locally, are usually sold to buyers from local markets. A fisher's earning is usually difficult to calculate due to a high variation in catches, but estimates are given for Búzios Island in 1987, where a fisher's average monthly earning ranged from 75 to 125 dollars (about two minimum wages) when the minimum salary in Brazil was 52 dollars. Another example is Ilha Comprida in 1999, where average earnings of fishers comprehended 212 dollars, also equivalent to roughly two minimum wages at that time [20].

Caiçaras live in areas relatively close to urban sites, such as Santos, São Paulo and Rio de Janeiro, but they live in areas designated for conservation, such as State and National Parks, or even the more legally restrict Biological or Ecological Reserves. Compared to the relatively isolated Amazonian rubber-tappers, Caiçaras have a low level of local organization in the management of resources [13]. Certainly, restrictions imposed by the Governmental Environmental Agencies have held a great impact on their subsistence and economy, since manioc cultivation is forbidden in many sites along with the prohibition of fishing in several rivers of the forest. An example of a decrease in the local capacity to maintain the internal control of the system, decreasing the social and ecological resilience of a community, influenced by legislation pressures, is ironically given by the relative unsuccessful management of Picinguaba, located inside the State Park of Serra do Mar, compared to the locally managed (community based management) beach of Almada and Engenho (Ponta do Almada), located outside the State Park boundaries [15].

## Methods

**Figure 1:**
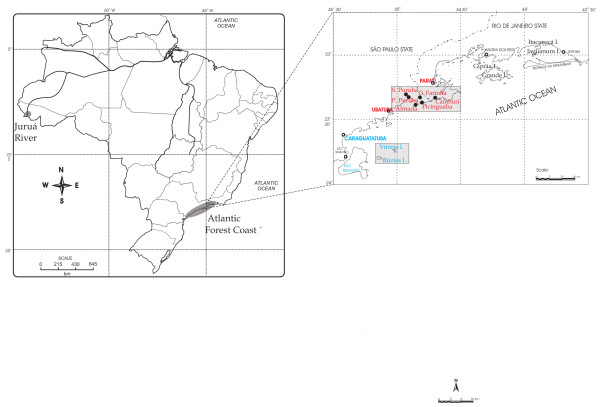
Study sites mentioned in the study, including metapopulations of *Caiçaras *(blue and red), inhabitants of the Atlantic Forest coast.

Data shown in this study were collected in different periods from 1986–1992 (Table 1). For this particular study, I show data collected through interviews, based on questionnaires. The procedures employed included visiting each house or family in each community, interviewing the resident couple. Interviewers included fisher residents, part-time and full time fishers, who comprehended the majority of the populations of the communities: islands of Búzios and Vitoria, and communities of Casa de Farinha, Puruba, Picinguaba, and Sertão do Puruba (Ubatuba district) (Figure 1). Questions asked in interviews, associated with this study, included place of born, earlier local of residence, and economic activities (Table 1). As already mentioned, there is published data on the Caiçaras, and the procedures in this study refer especially to the concepts and models used to analyze such data.

**Table 1:** Metapopulation I and II based on data on local migration (among islands and the coast).

**Locality**	**Number of people interviewed**	**Number born in the locality**	**Born in other localities Locality Name**	**Number of people born**
***Metapopulation I***				
**Búzios Island**	82	66		
**[1986]**			Vitória Island	7
			São Sebastião (city)	5
			São Sebastião Island	3
			Caraguatatuba	1
			Monte de Trigo Island	1
**Vitória Island**	16	11		
**[1992]**			Búzios Island	4
			São Sebastião Island	1
***Metapopulation II***				
**Casa de Farinha**	25	7		
**[1991]**			Ubatumirim	6
			Paratí	4
			Almada	2
			Picinguaba	2
			Ubatuba	2
			Puruba	1
			Joanópolis	1
**Picinguaba**	111	44		
**[1991]**			Parati	16
			Camburí	15
			Ubatuba	8
			Trindade	6
			Bahia*	4
			Almada	2
			Ceará*	2
			Praia da Fazenda	2
			Santos	2
			Ubatumirim	2
			Bebedouro	1
			Cunha	1
			Germany	1
			Maringá *	1
			Promirim	1
			São Paulo	1
			Sertão do Cambucá	1
			Terra Roxa	1
**Puruba Beach**	18	15		
**[1991]**			Ubatumirim	2
			Guarujá	1
**Sertão do Puruba**	43	16		
**[1991]**			Minas Gerais*	8
			Ubatuba	6
			Paraná*	2
			Paratí	2
			Bahia*	1
			Pernambuco	1
			Peruíbe	1
			Sertão do Cambucá	1

* Other State in Brazil

Less than 25 miles from the place of residence.

### Ecological concepts

Living in fragmented landscapes, such as the Atlantic Forest remnants, the Caiçaras of the SE Atlantic Forest are subject to processes of migration that seem to be relevant for their own survival. Ecological concepts, such as *metapopulation *and *resilience *are used in this study. Metapopulations are sets of subdivided populations ('populations of populations'), and much theory is credited to the classical publications by R. Levins in the seventies [32,33].

The understanding of the Caiçara as a metapopulation could help in the understanding of cultural exchanges due to local migration processes, which represents a source of cultural variation and of cultural diversity. Migration is a key point to the concept of metapopulation dynamics, since their persistence depends on the rate of dispersal among patches [34]. In this regard, the migration among the Caiçara populations should play an important role in their metapopulation dynamics. Populations are a central concern in evolution as they suffer the consequences of gene flow and of cultural trait diffusion. Models of coevolution of genes and culture have shown the population-level consequences in the diffusion of cultural traits [35]. Populations are the most basic unit of biodiversity dynamics [36] and, in case of local native populations, cultural influences are part of their dynamics, which are locally important but flow also regionally.

Metapopulation processes can be also associated to habitat change and to anthropic fragmentation of habitats. Therefore, it is a useful tool for biodiversity conservation, since predictions of fragmented landscapes depend upon analysis of the flow that occurs among the populations dispersed in the landscape. The metapopulation approach takes into consideration the fact that ecological processes occur in the local population and at metapopulation scales [32]. The sets of populations of Caiçaras, fragmented in the Atlantic Forest remnants, may be examples of human metapopulations, taking into account both genetic (through intermarriages) and cultural flows (between individuals and populations).

Resilience is the other ecological concept that might help thinking in the historical-ecological dynamic processes of the Caiçaras. The theory of adaptive cycles [37] is a tool to analyze resilience, including the processes of exploitation (rapid colonization), conservation (slow accumulation), release (creative destruction), and reorganization (minimal loss). There are three dimensions of the adaptive cycle: *potential for chan*ge (potential productivity and social or cultural potential, such as networks), *connectedness *(strength of internal connections that regulate internal processes and control external variability), and *resilience *(capacity of a system to experience disturbance and still maintain functions and controls) [38]. Resilience is the ability of societies to adapt to externally imposed changes [39], and there are examples of resilience analysis for large institutions, such as bureaucracies and industries in the literature [40]. For small-scale fishing communities in Brazil there are studies of the ecological and social resilience, including adaptive cycles, in the lagoon fishery of Ibiraquera, southern Brazil [41]. In the context of Caiçaras, such concepts help in understanding their historical process of change and adaptation, and in formulating hypotheses regarding possible outcomes.

## Results and discussion

In order to illustrate the analysis of the history, livelihood and interactions among the populations of Caiçaras, data on the northern coast of São Paulo will be shown in terms of a metapopulation and resilience analysis. Table 1 illustrates the migration processes between two sets of metapopulations: the populations of Búzios, Vitória Island and the coast, and the populations located close to the city of Ubatuba (Figure 1).

**Figure 2:**
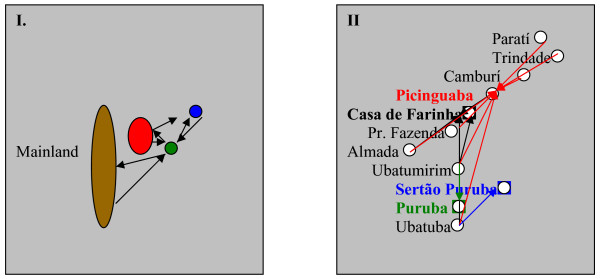
Metapopulation models of the Caiçaras from the northern coast of São Paulo, based on data in Table 1. Communities included are distant by less than 25 miles from the migrant's origin. *Metapopulation I *: brown: Mainland, (São Sebastião city and Caraguatatuba); red: São Sebastião Island or Ilhabela; green: Búzios Island; blue: Vitória Island. *Metapopulation II *: the arrows show the migration paths: black: Casa de Farinha; red: Picinguaba; blue: Sertão do Puruba; green: Puruba.

Metapopulation I includes migration between the mainland and the islands, in all directions. During the fieldwork, we gathered data on migration from Búzios Island to Ilhabela and to São Sebastião city (neighborhood of Bairro de Sao Francisco), as well as from Ilhabela to Búzios Island (Figure 2). Metapopulation II shows an intense interaction occurring among populations of the northern coast, between the districts of Ubatuba (São Paulo State) and Paratí (Rio de Janeiro State). In both cases, a spatially explicit metapopulation model is represented, assuming an inverse relationship of exchange (by migration) between populations and their distance [33]. That is, migration tends to concentrate between close or neighbor communities. The importance of such models is that they proportionate a representation of the level of connectedness among the populations of Caiçaras.

Connectivity is a measure of interaction or, in other words, of patch isolation, because migrants to a given patch decrease when the distance to their born place increases (Table 1) [32]. Note that migration processes bring together both genetic and cultural variations, and mixing in heterogeneous environments will increase the variance of initial phenotypes [35]. Migration is a strong cultural evolutionary force, since it brings cultural variability, which can be adaptive in changing or in heterogeneous environments. Such variability might have been important in order to proportionate the necessary flexibility to Caiçaras to deal with a changing environment, as the surroundings of the Atlantic Forest coast.

**Figure 3:**
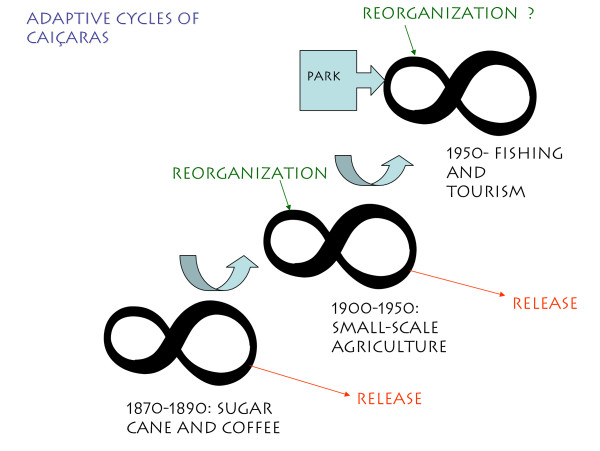
The adaptive cycles of caiçaras, considering the economic cycles of sugar cane and coffee (1870–1890), small-scale agriculture (1900–1950), and fishing and tourism (after 1950).

The degree of connectedness influences the internal and external processes of populations and as a part of adaptive cycles it is implicitly tied to resilience [38]. The adaptive cycles observed among the metapopulations of Caiçaras from the northern coast of São Paulo are shown in Figure 3. Such adaptive cycles are linked to historic and economic processes in the region, as observed in Table 2.

**Table 2:** Historic economic cycles on the Northern Coast of São Paulo

**Date**	**Activity**
Before 1870	Sugar cane plantations Brazilian rum
1870–1880	
Railroad built between Santos and São Paulo	Coffee plantations
	Manioc cultivated and commercialized
After 1890	Agriculture declined Isolation: small-scale agriculture concentrated on manioc.
After 1950	Decline of price of agricultural products: Activity concentrated on fishing: sand drums, weakfish [Sciaenidae], bluefish [Pomatomidae], mullets [Mugilidae], snooks, [Centropomidae], and shrimp, among others.
**After 1980**	**Fishing and the increase of tourism and tourist-related activities.** State Parks*: prohibition of small-scale agriculture and of fishing in rivers.

Source: [7, 17, 29]

*environmental legislation, State Park of Serra do Mar (*Parque Estadual da Serra do Mar*).

Associating the metapopulation of the Caiçaras (Figure 2) with their historical-ecological flow represented through the adaptive cycles in Figure 3, several questions can be posed:

**I) **Caiçaras adapted to different economic cycles through a relative flexibility in dealing with the environment and with the regional and national economy. They extracted local resources and participated in local and regional economy. Populations maintained their sizes, and maintained connections through migrations. Release was followed by reorganization in the economy towards a new cycle (Figure 3):

a. *How the restrictions imposed by the Serra do Mar State Park affect the Caiçaras' capacity of reorganization?*

b. Can such restrictions affect the resilience of the local populations, being an obstacle towards a new adaptive cycle?

c. If a new adaptive cycle does not occur, does it mean that a new stable state will not exist, and that the Caiçaras will loose their capacity to continue interacting with the forest resources?

d. Could a new adaptive cycle, based on tourism and on recreational fishing (the current tendency) be resilient, contributing to the ecological and social resilience of the Atlantic Forest and of the Caiçaras?

**II) **In metapopulations I and II (Figure 2), it may be suggested that the connectedness in genetic and cultural variants among and within the populations of Caiçaras is maintained through migrations among populations. In spite of their connectedness through metapopulations, the Caiçaras differ in the capacity of having strong communication channels within and among the metapopulations, compared to the experience of the caboclos, rural inhabitants of the Amazon [13,14,27]. For example, in the Amazon, The *Verdes Florestas Radio Station *(Upper Juruá Extractive Reserve), and the various communication systems in the Mamirauá Sustainable Development Reserve are examples of channels that integrate local residents and help in the decision processes concerning their environment and their economic activities. On the other hand, such lack of communication among Caiçaras does not imply that they could not be helpful in local management programs. The occurrence of local rules and practices, such as related to manioc cultivation and its local diversity [42,43] or related to the local fisheries, through an informal division in the use of fishing spots at sea, among others [2,19] are examples of behaviors that could flow through adequate communication channels.

a. In terms of communication channels, how does the relative slow connectedness of Caiçaras affect their capacity to overcome the impositions from the external system (legislation from the State Park, for example)?

b. How does the lack of communication channels among the Caiçaras decrease their empowerment capacity?

c. Are the external impositions (from legislation, for example), which exclude the local rules developed by the Caiçaras, already a result of a weak local power?

d. How does this weak local empowerment affect the Caiçaras' ecological and cultural resilience?

e. How will the extinction of the local Caiçaras affect the resilience of the last remnants of the Atlantic Forest coast?

## Brief conclusion

**Figure 4:**
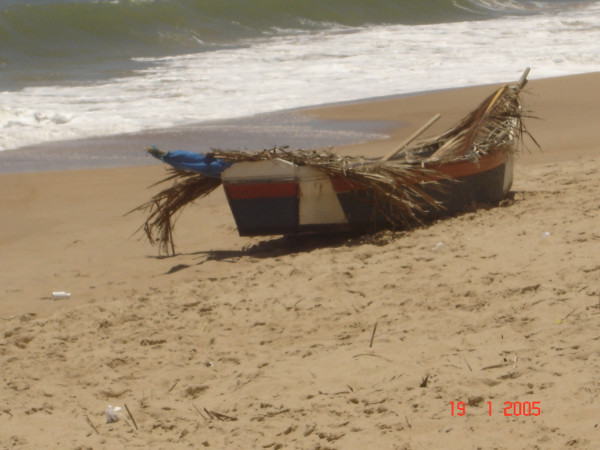
Fishing boat at Baixios, Bahia, after prohibition by governmental environmental agencies of constructing rafts using the wood *pau de jangada*.

The examples given here, describing the northern coast of São Paulo State, are part of the ecological-historical processes that occurs in the rest of the Brazilian coast. In Bahia, for example, during fieldwork in January 2005, I observed the last rafts used for fishing at Porto Sauípe, a community distant about 50 miles from Salvador. At neighboring communities, such as at Baixios, raft construction, according to local fishers, was forbidden by environmental agencies because of the use of *pau de jangada *(*Apeiba tibournou*) [44] to build the rafts. Fishers were trying to build differently shaped boats to continue fishing (Figure 4). In spite of the raft maneuverability on the rocky shores of Bahia and in other NE areas of Brazil, stressed in earlier studies[45] such a resilient feature is ignored by the environmental agencies. The highway that crosses the northern coast of Bahia, named *Estrada do Coco *(coconut highway), is reforested in some parts by exotic species, such as by *Pinus*. Would it not be wiser to include fishers in the management of the raft wood (*pau de jangada*) by planting such trees in available and suitable sites? Summing up, metapopulation analyses can be helpful in understanding interactions within these populations, and among populations and the environment. I am leaving many questions that could serve as a guide for future studies towards the sustainability of the last remnants of the Atlantic Forest coast and their inhabitants, the *Caiçaras*.
